# Body mass index and lung cancer risk in never smokers: a meta-analysis

**DOI:** 10.1186/s12885-018-4543-y

**Published:** 2018-06-05

**Authors:** Hongjun Zhu, Shuanglin Zhang

**Affiliations:** 1 Department of thoracic surgery, Shangqiu First People’s Hospital, Shangqiu, 476100 Henan China; 2 Department of Thoracic and Cardiovascular Surgery, the First Affiliated Hospital of Henan University, No. 357 Ximen Street, Kaifeng City, 475000 Henan Province China

**Keywords:** Lung cancer, Obesity, Risk factor, Smoking, Meta-analysis

## Abstract

**Background:**

Obesity is found to increase the risk of most cancer types, but reduce lung cancer risk in many studies. However, the association between obesity and lung cancer is still controversial, mainly owing to the confounding effect of smoking.

**Methods:**

Eligible studies were identified from electric databases to July 1, 2017. Relevant data were extracted and pooled using random-effects models; dose-response and subgroup analyses were also performed.

**Results:**

Twenty-nine studies with more than 10,000 lung cancer cases in15 million never smokers were included. Compared with normal weight, the summary relative risk (RR) was 0.77(95% confidence interval [CI]: 0.68–0.88, *P* < 0.01) for excess body weight (body mass index [BMI] ≥ 25 kg/m^2^). An inverse linear dose-response relationship was observed between BMI and lung cancer risk in never smokers, with an RR of 0.89(95% CI: 0.84–0.95, *P* < 0.01) per 5 kg/m^2^ increment in BMI. The results remained stable in most subgroup analyses. However, when stratified by sex, a significant inverse association existed in women but not in men. Similar results were found in analyses for other categories of BMI.

**Conclusion:**

Our results indicate that higher BMI is associated with lower lung cancer risk in never smokers.

**Electronic supplementary material:**

The online version of this article (10.1186/s12885-018-4543-y) contains supplementary material, which is available to authorized users.

## Background

Obesity is one of the most important risk factors for several major non-communicable diseases, including cardiovascular diseases, diabetes, and cancer, and the widespread prevalence of obesity is becoming a major threat to global public health [1]. Accumulating evidence suggest that excess body weight not only increases the overall cancer incidence but is also associated with worse outcomes in certain types of cancer [2–4].

As one of the most common cancers in both men and women, lung cancer causes more deaths than any other cancer [5]. Curiously, the association between obesity and lung cancer seems to be different from other cancer types, which has been disputed for years [6–9]. Many previous epidemiological studies found that higher body mass index (BMI) was associated with lower overall lung cancer risk, which was further confirmed in several meta-analyses [10–12] However, the results were always explained by the confounding effect of smoking, which was also associated with lower BMI [7]. Preclinical weight loss and socioeconomic status were also considered to be involved in the association [3, 13]. Hence, the true relationship between obesity and lung cancer risk remains to be clarified, and interpretation of data in only never smokers might be the best approach to reveal the real picture. Interestingly, several recent studies also reported that higher BMI was associated with better survival in patients with non-small cell lung cancer [14–16].

Lung cancer in never-smokers accounts for approximately 10–15% of all lung cancer patients and causes more than 15,000 deaths annually [17]. Concerning the association between obesity and lung cancer risk in never smokers, inconsistent results were also reported. In fact, subgroup analyses in previous meta-analyses have reported pooled results for the association. In the first meta-analysis performed by Yang Y, et al., found an significant inverse association between excess weight and lung cancer incidence in non-smokers based on 11 studies, while the association become insignificant for obesity and overweight categories [11]. Then Duan, et, al*.* also reported an attenuated linear dose-response association between BMI and lung cancer risk (including both incidence and mortality) in non-smokers, without statistical significance [12]. In the meta-analysis for lung cancer mortality by Shen N, et, al. in 2017, only 2 studies was included in subgroup analysis for never smokers, and the result was 0.95 (95%CI: 0.88–1.02) [10]. However, the results from the above three meta-analyses were sub-group analyses and based on only a small number of original studies included. To clarify the intrinsic association between obesity and the risk of lung cancer, and avoid the influence of confounding factors, we carried out an updated meta-analysis between body mass index and lung cancer risk in only never smokers, with a more complete literature search, which included both incidence and mortality to increase the sample size and statistical power.

## Methods

### Study selection

We searched the PubMed database to find relevant studies from January 1, 1966, to July 1, 2017.The following key words were used: *obesity*, *overweight*, *body mass index*, *body size*, *leanness*, or *anthropometric* in combination with *lung cancer*, *lung carcinoma*, or *lung neoplasm*. Our literature search was restricted to the full-text publications, and no language restriction was applied. The reference lists of identified articles and other similar meta-analyses were also checked to find additional studies.

### Eligibility criteria

Two independent investigators reviewed all the records and included studies that met the following criteria: 1) study population was never (or non-) smokers, current and former (past or ex-) smokers were not considered in this study, never smokers are defined as those who have not smoked greater than 100 cigarettes in their lifetimes and do not currently smoke; 2) the exposure of interest was BMI (kg/m^2^), including the categories of obesity, overweight, underweight or excess weight; 3) relative risk (RR) estimates (or hazard ratios or odds ratios) and 95% confidence intervals (CIs) for never smokers were reported or could be calculated from the data. 4) the outcome was the incidence or mortality of lung cancer; 5) observational studies with a cohort or case-control design. When duplicated studies were reported from the same population, the ones with the longest follow-up were included.

### Data extraction and quality assessment

For each study, the following data were extracted: the first author’s name, publication date, country, design, study population, BMI measurement, cancer ascertainment, sex, BMI categories with estimated midpoints, cases and participants per category, RRs with 95%CIs, and adjusted variables. RRs adjusted for the largest number of confounding variables were adopted. Quality of original studies was assessed by the Newcastle-Ottawa scale [18], which was widely used in observational studies, with a final score ≥ 7 considered as high quality.

### Statistical methods

Obesity and overweight were defined as BMI ≥30 and 25–29.99 kg/m^2^, in accordance with the definitions by of the World Health Organization, whereas excess weight combines the two categories. Normal weight was defined as 18.5–24.99 kg/m^2^, which was considered as the reference level. When the RRs with 95%Cis were reported by different BMI categories, the estimates for alternative comparisons were converted using the methods by Hamling et al. [19]. A fixed-effects model was employed to pool the results separated by sex. For some studies, we extracted the RR estimates from the figures presented in the manuscripts, using the software Engauge Digitizer version 2.11 (free software downloaded from http://sourceforge.net). A random-effects model was used to pool the individual RRs, considering the heterogeneity among studies, which was evaluated by the *Q* and *I*^2^ statistics [20].

Only studies that reported RRs with 95% CIs for at least 3categories were included into the dose-response analysis using the method proposed by Greenland [21] and Orsiniet al. [22]. For each BMI category, the average between the lower and upper boundary was assigned to the corresponding RR. When the extreme category was open-ended, the boundary was assumed to be the same amplitude as adjacent categories. RR trend estimates with 95%CIs in each study were calculated per 5 kg/m^2^increment in BMI and pooled together using a random-effects model. To compute the study-specific slope from the correlated log RR estimates across BMI levels, a two-stage generalized least-squares method with fractional-polynomial regression models was employed [22]. To test for nonlinearity, a likelihood ratio test was used to investigate the difference between nonlinear and linear models.

We also carried out subgroup analyses stratified by potential confounding factors, including study design, outcome, sex, diagnosis method, ethnicity, and quality. Meta-regression analyses were performed to explore the sources of heterogeneity. To evaluate the stability of the results, sensitivity analysis was employed to examine the change of pooled results after removing one study each time. Publication bias was assessed by funnel plot and Egger’s test, *p* < 0.10 was regarded as statistically significant, and the trim-and-fill method was used to adjust for potential bias. All statistical analyses were done with the STATA version 12.0 software (Stata Corporation, College Station, TX).

## Results

### Literature search and study characteristics

**Fig. 1:**
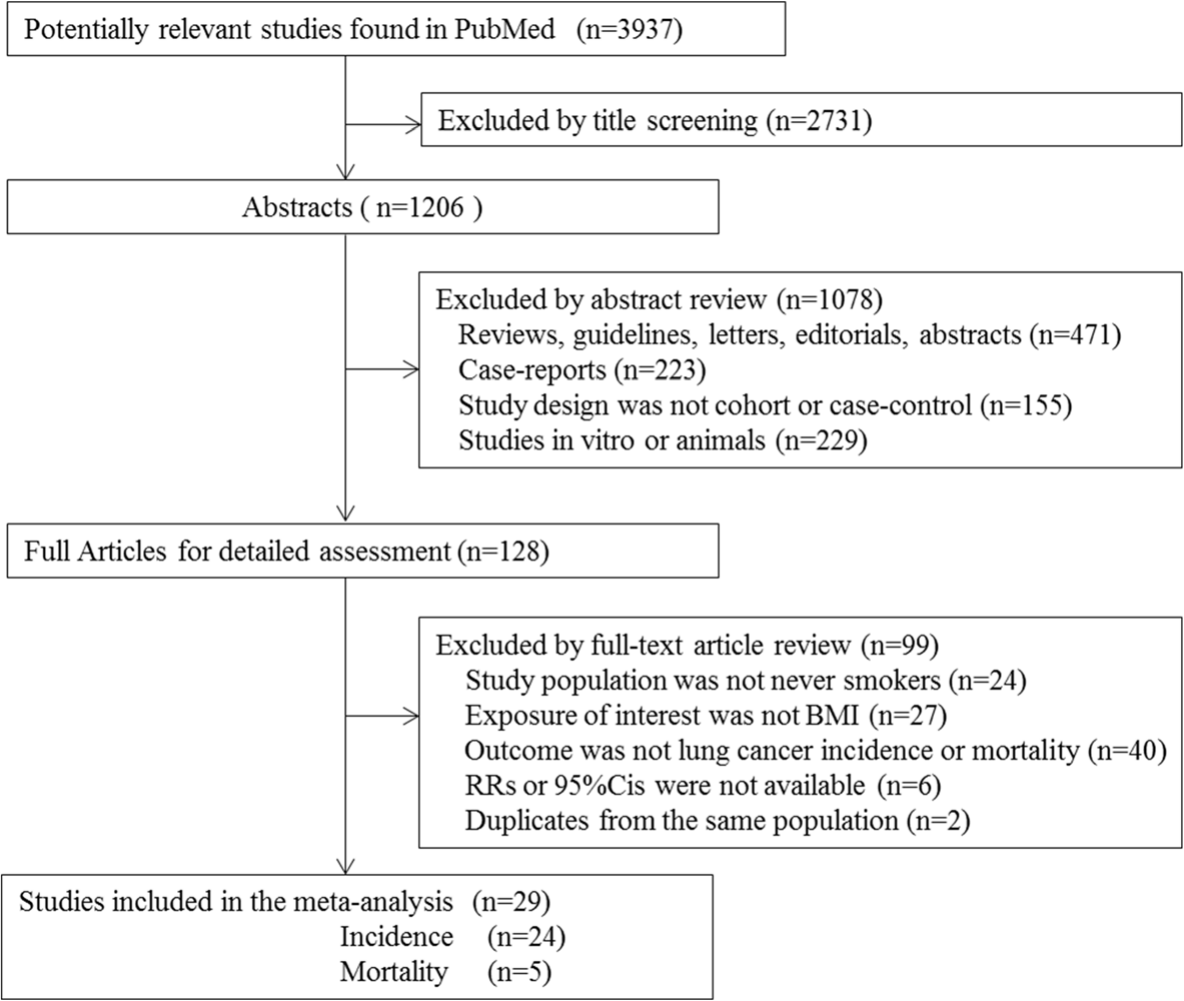
Flow chart of literature search

In total, 3937 articles were identified from the databases, and after removing the ineligible studies, 29 studies were included in the meta-analysis, including 21 cohort studies and 8 case-control studies (Fig. 1). Among these studies, 24 reported the RRs for lung cancer incidence and 5 provided mortality data. Twelve were from America, 7 from Europe, 10 from Asia. Two studies in Chinese were included in our study [23, 24]. 2studies were excluded because of multiple reports of the same population [7, 25].

In general, the quality scores ranged from 4 to 9 with an average of 7 points (7.3 for cohort studies and 6.2 for case-control studies). Among all studies, 21 were considered as high quality (≥7 points). The most common confounders adjusted in original studies included age, sex, alcohol consumption, vegetable/fruit intake, and physical activity; however, few studies were controlled for total calorie intake, other chronic diseases, concomitant medication, or environmental status. The baseline characteristics of all studies are shown in Additional file 1: Table S1 and the quality scores are listed in Additional file 2: Table S2 and Additional file 3: Table S3.

### Overall analyses

**Fig. 2:**
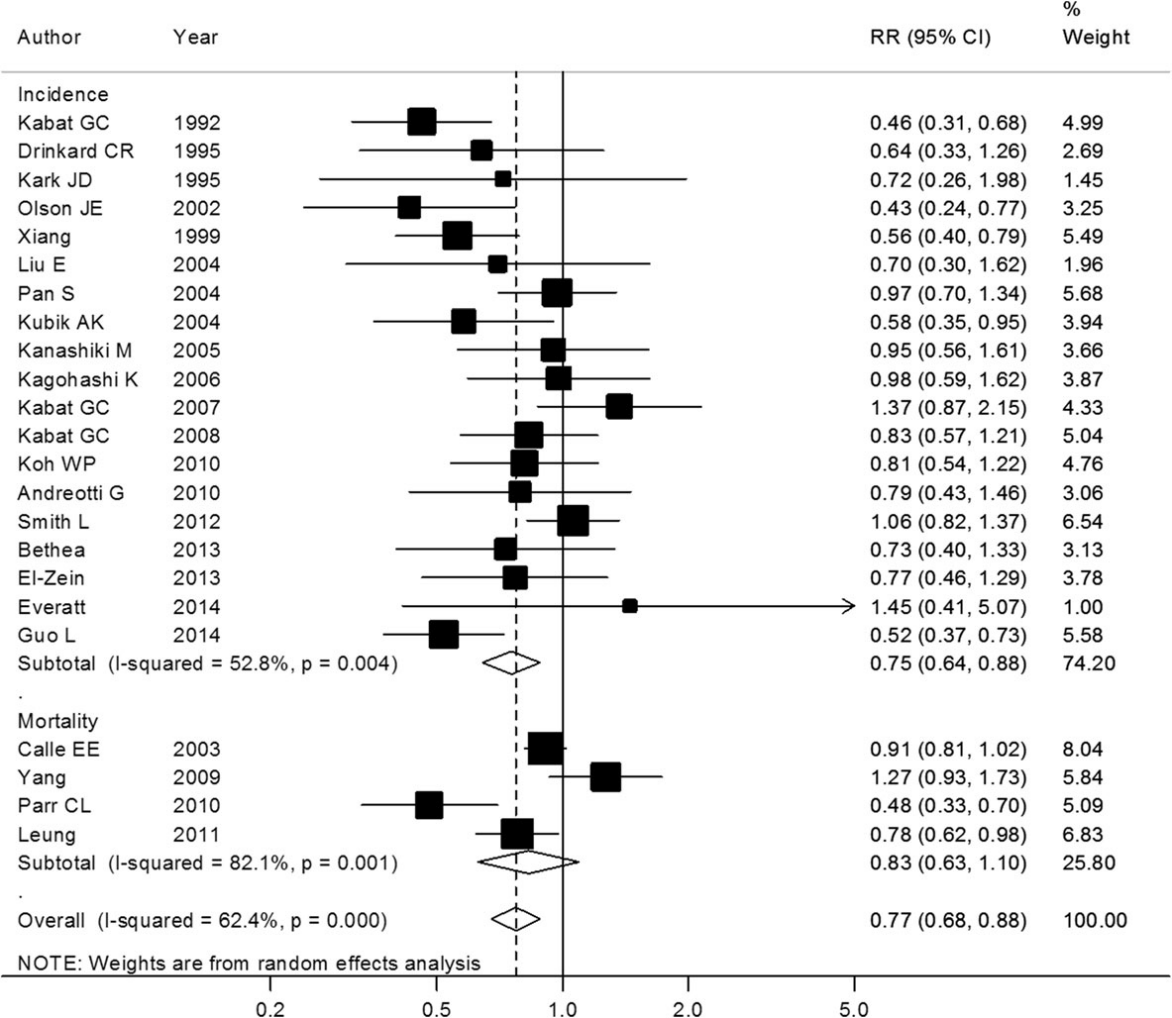
Excess weight and lung cancer risk in never smokers. Box sizes reflect the weights of studies included in the meta-analysis, horizontal lines are the 95% CIs, and the summary RR is represented by the diamond. RR: relative risk, CI: confidence interval

Overall analyses showed that there was an inverse association between BMI and lung cancer risk in never smokers. Eighteen and 15 studies reported the data for obesity and overweight categories, respectively. After pooling all results, RRs were 0.78(95% CI: 0.65–0.94, *P* = 0.01) and 0.76(95% CI: 0.65–0.87, *P* < 0.01) compared with the normal category. Combined analysis of 23 studies showed that the RR was 0.77(95% CI: 0.68–0.88, *P* < 0.01) for the excess weight category (Fig. 2). Substantial heterogeneity was observed among the included studies, *I*^2^ was 54.30, 50.60, and 62.40% for obesity, overweight, and excess weight categories, respectively.

Subgroup analyses suggested that RRs did not differ significantly by design, outcome, cancer ascertainment, BMI assessment, quality, or whether important confounders were adjusted for in original studies, although some results were statistically negative, mainly owing to the small number of studies included. When stratified by sex, some differences were observed; the results for women were consistently significant for all categories, whereas no positive associations were found for men (Table 1). To avoid the disturbance by preclinical weight loss caused by early lung cancer itself, studies were ruled out in which BMI was measured < 5 years before the diagnosis of lung cancer [12, 23, 25–28], and the pooled analysis of remaining studies gave an RR of 0.79 (95% CI: 0.70–0.91, *P* < 0.01) for the excess weight category.

**Table 1:** Subgroup analyses for the association between BMI and lung cancer risk in never smokers

Categories	Subgroups	Number of studies	RR (95% CI)	*P* value	Heterogeneity			*P-interaction*
					chi-squared	*I* ^*2*^	*P-heterogeneity*	
Obesity		18	0.78(0.65–0.94)	0.01	37.23	54.30%	< 0.01	
Design	Cohort	14	0.74(0.60–0.91)	< 0.01	27.83	53.30%	0.01	0.32
	Case-control	4	0.97(0.58–1.62)	0.90	8.75	65.70%	0.03	
Outcome	Incidence	15	0.79(0.63–0.99)	0.04	30.83	54.60%	< 0.01	0.74
	Mortality	3	0.71(0.45–1.12)	0.14	6.40	68.70%	0.04	
Gender	Male	5	0.69(0.41–1.15)	0.16	16.58	69.80%	< 0.01	0.81
	Female	8	0.86(0.72–1.02)	0.08	7.49	19.90%	0.28	
Ethnicity	Non-Asian	13	0.82(0.64–1.04)	0.10	27.92	57.00%	< 0.01	0.51
	Asian	5	0.70(0.54–0.91)	< 0.01	5.56	28.00%	0.24	
Quality	High	12	0.76(0.61–0.94)	0.01	27.5	60.00%	0.004	0.72
	Low	6	0.84(0.55–1.29)	0.43	9.72	48.60%	0.08	
Diagnosis	Registry	10	0.77(0.59–1.00)	0.05	22.28	59.60%	< 0.01	0.89
	Pathology	8	0.79(0.59–1.06)	0.11	14.16	50.60%	0.05	
Adjustment for confounders								
Alcohol intake	Yes	11	0.83(0.68–1.02)	0.07	18.98	47.30%	0.04	0.49
	No	7	0.68(0.45–1.04)	0.07	16.25	63.10%	0.01	
Vegetable/fruit intake	Yes	6	0.81(0.58–1.13)	0.22	12.80	60.90%	0.02	0.80
	No	12	0.76(0.59–0.97)	0.03	23.41	53.00%	0.02	
Physical activity	Yes	9	0.91(0.75–1.11)	0.34	12.18	34.30%	0.14	0.19
	No	9	0.67(0.49–0.93)	0.02	18.15	55.90%	0.02	
Medical history^a^	Yes	3	0.78(0.58–0.95)	0.24	35.80	60.90%	< 0.01	0.61
	No	15	0.90(0.76–1.07)	0.02	0.78	0.00%	0.68	
Overweight		15	0.76(0.65–0.87)	< 0.01	28.37	50.60%	0.01	
Design	Cohort	12	0.74(0.62–0.88)	< 0.01	27.30	59.70%	< 0.01	0.78
	Case-control	3	0.80(0.63–1.02)	0.08	1.03	0.00%	0.60	
Outcome	Incidence	12	0.76(0.64–0.90)	< 0.01	16.92	35.00%	0.11	0.93
	Mortality	3	0.73(0.53–1.01)	0.06	10.51	81.00%	< 0.01	
Gender	Male	5	0.92(0.62–1.36)	0.68	14.38	72.20%	< 0.01	0.44
	Female	8	0.82(0.72–0.93)	< 0.01	6.52	0.00%	0.48	
Ethnicity	Non-Asian	11	0.87(0.78–0.97)	< 0.01	10.34	3.30%	0.41	0.09
	Asian	4	0.61(0.44–0.86)	< 0.01	10.87	72.40%	0.01	
Quality	High	10	0.85(0.77–0.94)	< 0.01	9.47	4.90%	0.40	0.32
	Low	5	0.70(0.46–1.06)	0.09	13.76	70.90%	< 0.01	
Diagnosis	Registry	8	0.82(0.68–0.98)	0.03	14.38	51.30%	0.04	0.25
	Pathology	7	0.68(0.55–0.84)	< 0.01	8.74	31.40%	0.19	
Adjustment for confounders								
Alcohol intake	Yes	11	0.79(0.68–0.92)	< 0.01	18.32	45.40%	0.05	0.37
	No	4	0.67(0.46–0.97)	0.03	6.36	52.80%	0.10	
Vegetable/fruit intake	Yes	5	0.89(0.79–0.99)	0.03	1.46	0.00%	0.83	0.15
	No	10	0.68(0.54–0.85)	< 0.01	22.26	50.60%	< 0.01	
Physical activity	Yes	9	0.87(0.77–0.97)	0.02	8.86	9.70%	0.35	0.14
	No	6	0.66(0.49–0.88)	< 0.01	12.85	61.10%	0.02	
Medical history^a^	Yes	3	0.89(0.79–1.00)	0.05	24.35	0.00%	0.66	0.42
	No	12	0.72(0.60–0.87)	< 0.01	0.84	50.60%	0.01	
Excess weight		23	0.77(0.68–0.88)	< 0.01	58.46	62.40%	< 0.01	
Design	Cohort	16	0.80(0.69–0.94)	< 0.01	40.07	62.60%	< 0.01	0.47
	Case-control	7	0.71(0.56–0.91)	< 0.01	13.78	56.50%	0.03	
Outcome	Incidence	19	0.75(0.64–0.88)	< 0.01	38.12	52.80%	< 0.01	0.60
	Mortality	4	0.83(0.63–1.10)	0.19	16.73	82.10%	< 0.01	
Gender	Male	8	0.88(0.70–1.11)	0.29	17.94	55.40%	0.02	0.85
	Female	12	0.75(0.62–0.91)	< 0.01	28.89	61.90%	< 0.01	
Ethnicity	Non-Asian	14	0.80(0.68–0.95)	< 0.01	28.25	54.00%	< 0.01	0.66
	Asian	9	0.74(0.59–0.94)	0.01	26.05	69.30%	< 0.01	
Quality	High	16	0.83(0.73–0.95)	< 0.01	30.06	50.10%	0.01	0.18
	Low	7	0.68(0.50–0.90)	< 0.01	17.7	66.10%	< 0.01	
Diagnosis	Registry	11	0.88(0.75–1.05)	0.15	24.27	58.80%	< 0.01	0.06
	Pathology	12	0.68(0.57–0.81)	< 0.01	19.97	44.90%	0.05	
Adjustment for confounders								
Alcohol intake	Yes	12	0.83(0.71–0.97)	0.02	26.74	58.90%	0.01	0.31
	No	11	0.71(0.57–0.89)	< 0.01	22.73	56.00%	< 0.01	
Vegetable/fruit intake	Yes	7	0.85(0.69–1.05)	0.13	17.29	57.10%	< 0.01	0.37
	No	16	0.73(0.62–0.87)	< 0.01	34.93	65.30%	< 0.01	
Physical activity	Yes	9	0.87(0.77–0.99)	0.04	10.14	21.10%	0.26	0.52
	No	14	0.75(0.61–0.92)	< 0.01	41.15	68.40%	< 0.01	
Medical history^a^	Yes	7	0.84(0.68–1.04)	0.11	13.32	55.00%	0.04	0.52
	No	16	0.75(0.63–0.89)	< 0.01	40.57	63.00%	< 0.01	
BMI increase per 5 Kg/m^2^		28	0.89(0.84–0.95)	< 0.01	86.44	68.80%	< 0.01	
Design	Cohort	20	0.89(0.82–0.96)	< 0.01	58.12	67.30%	< 0.01	0.88
	Case-control	8	0.90(0.79–1.03)	0.13	24.77	71.70%	0.01	
Outcome	Incidence	23	0.89(0.83–0.96)	< 0.01	65.3	66.30%	< 0.01	0.98
	Mortality	5	0.89(0.76–1.04)	0.16	20.98	80.90%	< 0.01	
Gender	Male	12	0.96(0.83–1.11)	0.60	28.43	61.30%	< 0.01	0.42
	Female	13	0.89(0.81–0.97)	< 0.01	34.74	65.50%	< 0.01	
Ethnicity	Non-Asian	18	0.91(0.84–0.98)	0.01	47.23	64.00%	< 0.01	0.66
	Asian	10	0.88(0.78–0.98)	0.03	33.12	72.80%	< 0.01	
Quality	High	20	0.91(0.86–0.98)	< 0.01	51.74	63.30%	< 0.01	0.39
	Low	8	0.85(0.72–1.00)	0.05	26.41	73.50%	< 0.01	
Diagnosis	Registry	15	0.93(0.86–1.00)	0.06	42.41	67.00%	< 0.01	0.35
	Pathology	13	0.86(0.78–0.95)	< 0.01	32.26	62.80%	< 0.01	
Adjustment for confounders								
Alcohol intake	Yes	15	0.89(0.83–0.95)	< 0.01	36.97	62.10%	< 0.01	0.60
	No	13	0.92(0.79–1.07)	0.28	48.42	75.20%	< 0.01	
Vegetable/fruit intake	Yes	7	0.93(0.84–1.02)	0.13	12.57	52.3%	0.05	0.77
	No	21	0.89(0.82–0.96)	< 0.01	72.90	72.6%	< 0.01	
Physical activity	Yes	10	0.92(0.86–0.98)	< 0.01	11.33	20.60%	0.25	0.96
	No	18	0.90(0.81–0.99)	< 0.01	86.44	77.3%	< 0.01	
Medical history^a^	Yes	11	0.90(0.83–0.97)	< 0.01	29.05	65.60%	< 0.01	0.84
	No	17	0.88(0.80–0.98)	0.02	56.45	71.70%	< 0.01	

Note**:** In the subgroup analyses, ^a^medical history included history of chronic lung disease, history of family lung cancer, diabetes status, and hormone treatment. Other confounders such as total energy intake, environmental status and concomitant medication (use of aspirin and metformin) were not common in original studies; thus, subgroup analyses stratified by them were not performed

### Dose-response analyses

**Fig. 3:**
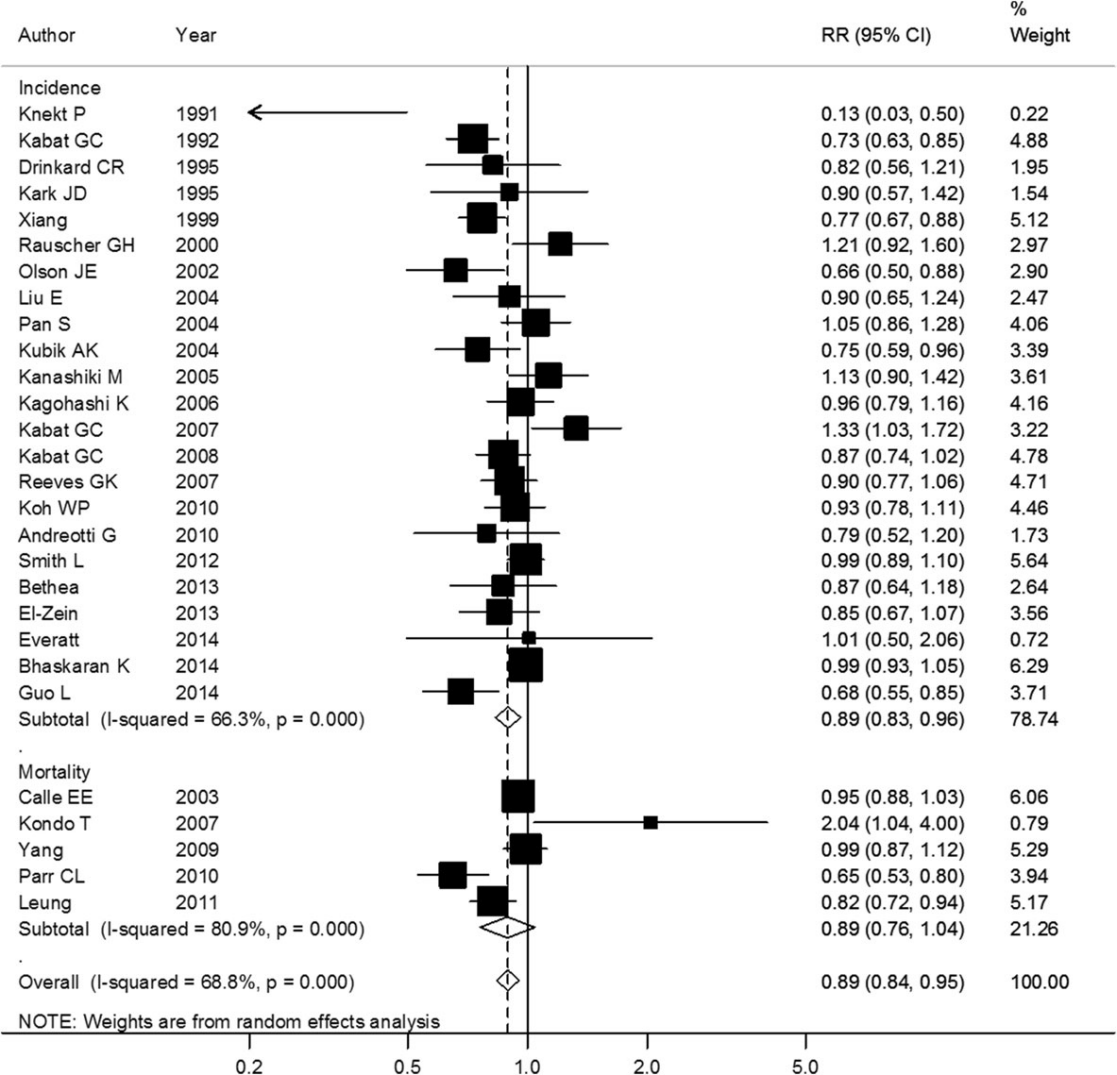
Association between BMI and lung cancer risk per 5 kg/m^2^ increase. Box sizes reflect the weights of studies included in the meta-analysis, horizontal lines are the 95% CIs, and the summary RR is represented by the diamond. BMI: body mass index, RR: relative risk, CI: confidence interval

**Fig. 4:**
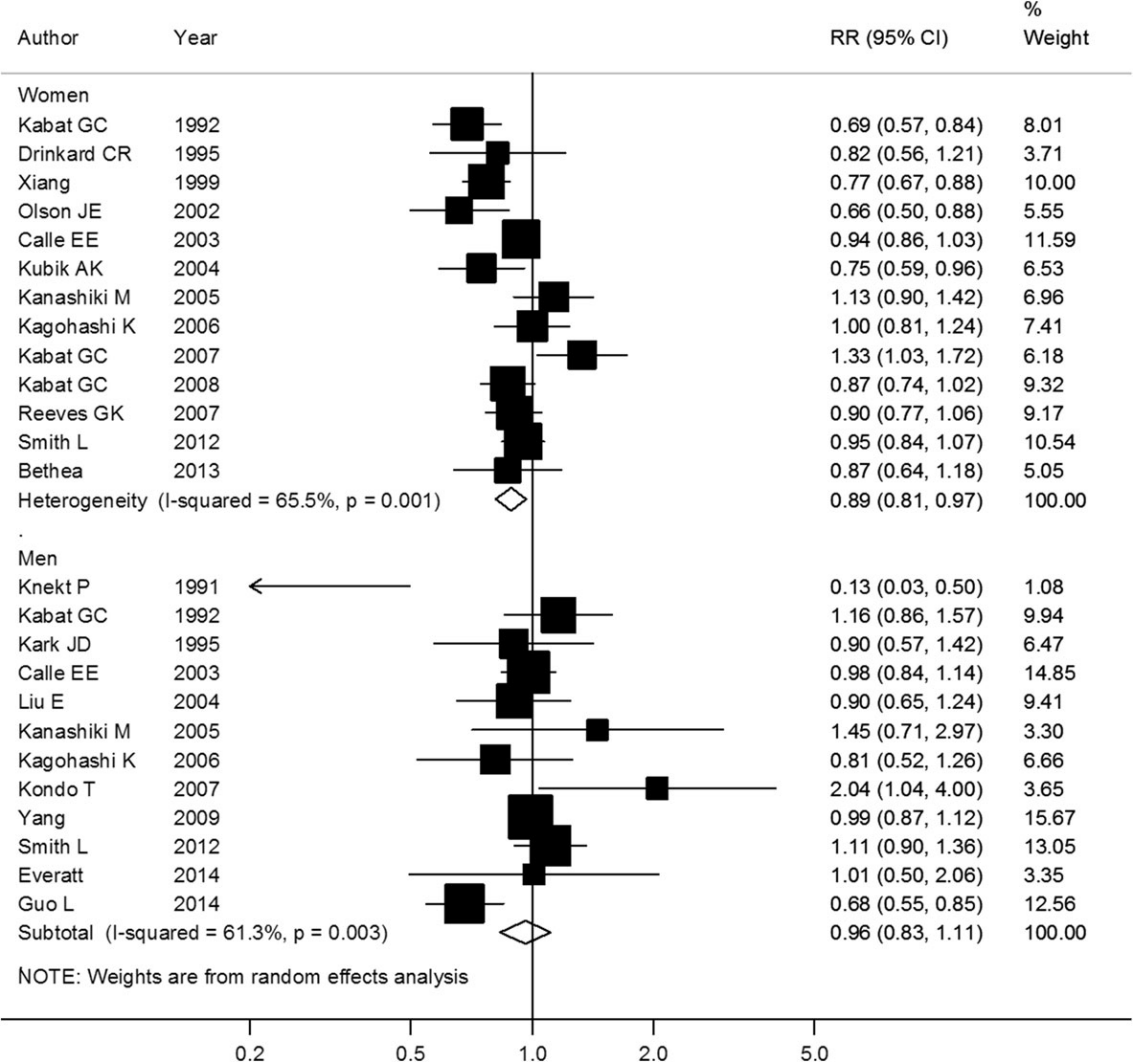
Association between BMI and lung cancer risk per 5 kg/m^2^ increase, stratified by sex. Box sizes reflect the weights of studies included in the meta-analysis, horizontal lines are the 95% CIs, and the summary RR is represented by the diamond. BMI: body mass index, RR: relative risk, CI: confidence interval

Finally, 28 studies were included in the dose-response analysis; the summary RR was 0.89(95% CI: 0.84–0.95, *P* < 0.01) per 5 kg/m^2^ increase in BMI, with a high heterogeneity (*I*^2^ = 86.44%) (Fig. 3).The RRs were 0.89(95% CI: 0.82–0.96, *P* < 0.01) for cohort studies and 0.90(95% CI: 0.79–1.03, *P* = 0.13) for case-control studies (Table 1). No significant differences were observed in subgroup analyses stratified by most confounders. When stratified by sex, the RRs were 0.89(95% CI: 0.81–0.97, *P* < 0.01) and 0.96(95% CI: 0.83–1.11, *P* = 0.60) for women and men, respectively (Fig. 4). The combined analysis of studies in which BMI was measure > 5 years before diagnosis gave an RR of 0.90 (95% CI: 0.84–0.96, *P* < 0.01) per 5 kg/m2 increase in BMI.

**Fig. 5:**
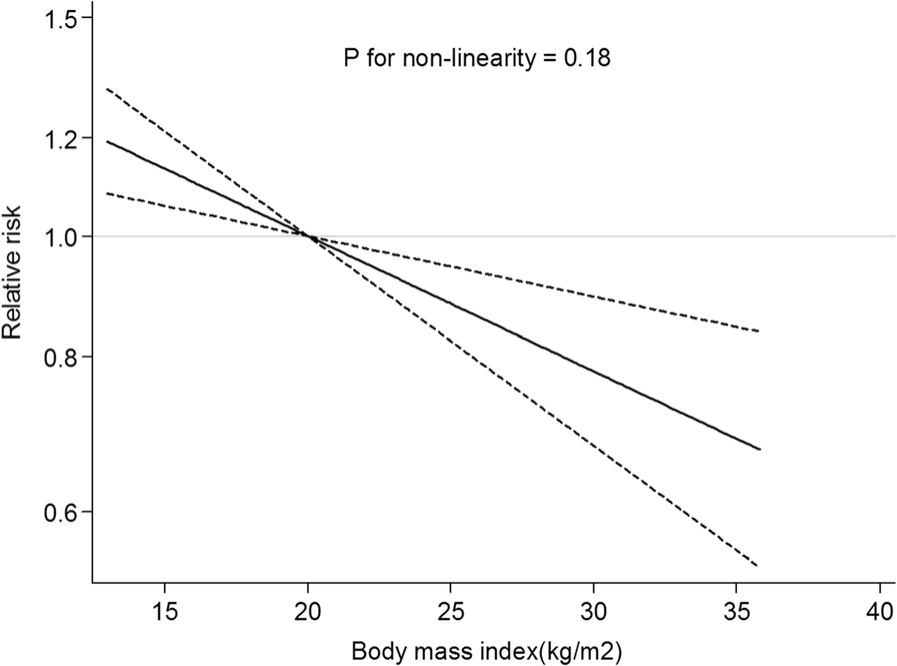
Dose-response analysis for body mass index and lung cancer risk in never smokers. The solid line represents the trend between BMI and lung cancer risk, and the dashed lines represent the 95% confidence intervals. The displayed *p*-values refer to the test for nonlinearity

No evidence of a nonlinear relationship between BMI and lung cancer risk in never smokers was found (*p* for nonlinearity = 0.18), and an inverse linear trend was fitted in a random-effects meta-regression model (Fig. 5). Compared with BMI of 20 kg/m^2^, the RRs were 1.17(95% CI: 1.02–1.34, *P* = 0.02), 0.87(95% CI: 0.83–0.92, *P* < 0.01), 0.81(95% CI: 0.76–0.87, *P* < 0.01), and 0.80 (95% CI: 0.68–0.94, *P* < 0.01) for BMI of 15, 25, 30, and 35 kg/m^2^, respectively. When stratified by sex, the inverse linear trend was still present for women but disappeared for men.

### Meta-regression, sensitivity analyses, and publication bias

As described above, substantial heterogeneity was observed across studies, but meta-regression analyses showed that most of the confounders including BMI assessment, ethnicity, design, quality, outcome, and diagnosis method were not significantly associated with the heterogeneity. After excluding the 2 outlier studies by Kabat*, et, al.* [29] and Kondo, *et, al.* [30], the heterogeneity was reduced to some extent, but the results were unchanged. The results were still robust after removing one specific study each time in the sensitivity analysis. A slight publication bias was found in the analysis of the obesity category (*p* = 0.046 by Egger’s test, *p* = 0.20 by Begg’s test), but no studies were needed to be filled with the use of trim and fill method, suggesting that the influence could be negligible. In fact, the bias might be caused by insufficient data reported in original studies on the category of obesity, since in other category analyses, the funnel plots seemed to be symmetrical, and no significant publication biases were found.

## Discussion

In the pooled analysis of 29 observational studies, involving more than 10,000 lung cancer cases in 15 million never smokers, the results suggested that higher BMI was associated with lower lung cancer risk, especially in women. In contrast with previous meta-analyses, our study includes the largest sample up to now, and the results were stable both in the subgroup and dose-response analyses.

Previous studies reported that obesity was associated with a lower risk of certain cancer types, particularly smoking related-cancers [2, 11, 12, 31]. However, the results were less convincing owing to the small sample size and other confounding factors, especially smoking. Meta-analysis is a quantitative approach that combines the results from multiple studies and increases the statistical power to resolve uncertainty in single studies for a more reliable conclusion. Thus, our study has a number of advantages. We included all the eligible epidemiological studies investigating the association between BMI and lung cancer risk in never smokers and redefined comparable exposure categories, which allowed for better control of confounders, subgroup analyses, and further dose-response analyses.

As we mentioned previously, several hypotheses have been put forward to explain the inverse association between lung cancer risk and BMI. As our study was limited to never smokers, the confounding of smoking, one of the most common arguments for the trend, was avoided as much as possible. A second hypothesis is that the lower BMI might reflect preclinical weight loss caused by early lung cancer itself or other related diseases. To solve this doubt, only studies in which BMI was measured5 or more years before diagnosis were analyzed, and the inverse association remained unchanged. In fact, the participants with previous clinical weight loss were excluded at recruitment in most studies. It was also speculated that socioeconomic factors might be relevant to the inverse association, such as indoor air pollution in developing countries; however, our results indicated that the inverse association was stable across the strata of ethnicity.

Interestedly, we found a sex difference in the association between BMI and lung cancer risk, although p-interaction for sex is not statistically significant. Previous studies reported paradoxical results concerning the differences between sexes [11, 12], Smith *et al.* found that BMI was more strongly related to lower lung cancer risk in women than in men in a large cohort study [26]. The authors speculated that estrogens might play a protective role in lung cancer development [26], which was in accordance with our results. Notably, since more men than women are smokers, the results for the men in our meta-analysis might be influenced by the relatively smaller sample size, more studies are warranted to explore the sex difference.

Not only epidemiological studies revealed the possibility that obesity might lower lung cancer risk, but some biological discoveries also provided useful hints. It has long been noted that lower BMI might increase the susceptibility of DNA to chemical carcinogens in cigarettes [27], and excess weight was associated with decreased chromosome damage [28]. Some studies also suggested that adipose tissue was helpful to keep the memory of CD8^+^ T cells to maintain normal immune functions [32]. In addition, increased insulin-like growth factor-1 level which might explain the obesity-carcinogenesis connection was found not to be associated with lung cancer [33]. Paradoxically, systemic inflammation, which might increase lung cancer risk [33], is also closely associated with obesity [34]. These studies provide possible direction for more in-depth research in this field to better clarify the obesity paradox in lung cancer development.

The results of our study should be interpreted with caution, as associations found in a meta-analysis of observational studies do not reveal causation. Several other limitations should also be considered. First, concerning the outcomes of interest, both the incidence and mortality of lung cancer were included. It is reasonable to do this since lung cancer is relatively rare in the overall population, and previous studies reported that the incidence and mortality of lung cancer almost coincided with each other [35]. Second, inherent limitations in original studies were inevitable, especially for the case-control studies, which were prone to recall and selection bias, inconsistencies in baseline characteristics of original studies including different study populations, pathology type, ethnicity, ages, and duration all contributed to heterogeneity across studies. However, no individual confounder significantly influenced the heterogeneity by meta-regression, and subgroup analyses by these confounders were almost the same, indicating the stability of our results. Third, in some studies, BMI was calculated by self-reported weight and height, and different exposure ranges were adopted across different studies, which might lead to some incomparability of results. To solve the problem, we conducted different category comparisons and dose-response analyses as well as subgroup analyses stratified by BMI assessment, and the results were consistently stable to support our conclusion, which was further validated by the sensitivity analyses In addition, although no significant differences were found between studies whether they were adjusted for common confounders or not, insufficient adjustment of other potential confounders, including secondhand smoking, occupational exposure to lung carcinogens (e.g., radon), concomitant medication (e.g., using of aspirin and metformin) might distort our results, and we also had insufficient data on different histological types of lung cancer for further subgroup analyses. Lastly, only articles in full-text were included in our analysis, abstracts, trial registries were not retrieved, some studies might be missed. However, no significant publication bias was observed except in the analysis of the obesity category, and no studies were required using the “trim and fill” method, suggesting that the influence was slight.

Every sword has two edges. Obesity has been stereotyped as a risk factor for many chronic diseases including most types of cancer, but our study shows that higher BMI is associated with lower lung cancer risk, especially in women. Our results do not suggest increasing body weight to decrease the risk of lung cancer; however, underweight is also inadvisable, and maintaining a proper weight is the best choice. The results of our study are helpful to explain the J-shaped association between BMI and total mortality [1]. Further studies should be focused on the mechanisms underlying the phenomena, and extra efforts are needed to reduce the unfavorable effects of obesity on most cancer types and other chronic diseases.

## Conclusions

In conclusion, the results of our meta-analysis indicate that higher BMI is associated with lower lung cancer risk, especially in women, which alter our common understanding of the relationship between obesity and cancer, although the causal relationship between these two factors cannot be determined from this analysis. Additional studies are required to validate these findings and to better understand the biologic rationale for this observation.

## Additional files

Additional file 1: **Table S1.** Characteristics of studies included in the meta-analysis of obesity and lung cancer risk in non-smokers. (DOCX 88 kb)

Additional file 2: **Table S2.** Quality scores of the cohort studies included in the meta-analysis, assessed by the Newcastle-Ottawa scale. (DOCX 20 kb)

Additional file 3: **Table S3.** Quality scores of the case-control studies included in the meta-analysis, assessed by the Newcastle-Ottawa scale. (DOCX 18 kb)

## Abbreviations

BMI: body mass index
CI: confidence interval
FTO: fat mass- and obesity-associated gene
RR: relative risk
