# Supplementary material for: Body mass index and lung cancer risk in never smokers: a meta-analysis
Source: BMC Cancer. 2018 Jun 5;18:635. doi: 10.1186/s12885-018-4543-y (PMC5987408; doi:10.1186/s12885-018-4543-y)
Supplement: Supplementary file 1 — Table S1. Characteristics of studies included in the meta-analysis of obesity and lung cancer risk in non-smokers. (DOCX 88 kb) [file 12885_2018_4543_MOESM1_ESM.docx]

**Table S1 Characteristics of studies included in the meta-analysis of obesity and lung cancer risk in non-smokers**

| **Author,**  **Year, Ref** | **Dura-tion** | **Study population** | **Design** | **Out- come** | **BMI**  **assessment** | **Cancer ascertain- ment** | **Sex** | **Cases per**  **categ-ory** | **Particip-ants per category‡** | **Categories** | **Mid- points** | **RRs** | **Adjustment for**  **confounding factors** |
| --- | --- | --- | --- | --- | --- | --- | --- | --- | --- | --- | --- | --- | --- |
| Knekt P  1991, Finland[^1^](#_ENREF_1) | 1966-1984 | Finnish men examined by the Social Insurance Institution's Mobile Clinic | Cohort | Inci | Measured at the baseline | Registry | F | 17 | 6842 | ≤22.5  >27.0 | 18.75  31 | 7.6(2.0-29.4)  1.00(ref) | Age, intake of different nutrients and foods. |
| Kabat GC  1992, USA[^2^](#_ENREF_2) | 1981-1990 | Patients in 28 hospitals in eight US cities | HCC | Inci | Self-reported at 5 years prior to diagnosis | Pathology | F  M | 48  37  23  19  11  13  23  22 | 444  413  316  501  173  427  542  462 | <22  22-24.9  25-27.9  ≥28  <22  22-24.9  25-27.9  ≥28 | 18.5  23.45  26.45  31.5  18.5  23.45  26.45  31.5 | 2.90(1.6-5.00)  2.40(1.30-4.20)  1.90(0.90-6.50)  1.00(ref)  0.90(0.40-2.10)  0.50(0.30-1.10)  0.80(0.40-1.50)  1.00(ref) | Age, education, race, hospital, time period, alcohol Intake, and history of chronic lung disease. |
| Drinkard CR  1995, USA[^3^](#_ENREF_3) | 1986-1992 | Women with a valid Iowa driver's license | Cohort | Inci | Self-reported at baseline | Registry | F | 15  10  11 | *712882928206* | < 24.31  24.31-28.38  > 28.38 | 21.4  26.34  31.69 | 1.00 (ref)  0.61 (0.27-1.38)  0.68 (0.31-1.52) | Age,education, physical activity, and beer intake. |
| Kark JD  1995, Israel[^4^](#_ENREF_4) | 1963-1986 | the Israel Civil Servant Study | Cohort | Inci | Measured at baseline | Pathology | M | 6  4  18 | 762  931  3104 | ≤22.93  22.93-24.81  >24.81 | 18.95  23.87  29.9 | 1.00(ref)  0.55(0.16-1.90)  0.72(0.26-1.96) | Age and city. |
| Xiang  1999, China[^5^](#_ENREF_5) | 1992-1993 | Non-smoking women in Shanghai China | PCC | Inci | Self-reported at 5 years prior to diagnosis | Pathology | F | 94  103  129  176 | 152  159  140  150 | ≥25.15  22.86-25.14  20.96-22.85  <20.96 | 30.08  24  21.90  17.98 | 1.00(ref)  1.02(0.71-1.47)  1.50(0.97-2.01)  1.79(1.26-2.55) | Age, education, economy income, intake of different nutrients, tuberculosis and lung cancer family history, menopausal status. |
| Rauscher GH  2000, USA [^6^](#_ENREF_6) | 1982-1985 | People in New York State | PCC | Inci | Self-reported prior illness | Pathology | B | 23  137  28 | 35  133  17 | ≤21.26  21.26-30.84  >30.84 | 19.88  26.05  35.42 | 1.0(ref)  1.2(0.6-2.1)  1.8(0.8-4.5) | Sex and educational attainment, preclinical weight loss in the present study. |
| Olson JE  2002, USA [^7^](#_ENREF_7) | 1986-1998 | The Iowa Women’s Health Study | Cohort | Inci | Self-reported at baseline | Pathology | F | 15  19  13  10  16 | *42195011512051895290* | ≤22.89  22.90-25.04  25.05-27.43  27.44-30.69  ≥30.70 | 20.7  23.97  26.24  29.06  32.85 | 1.00(ref)  0.82(0.43-1.57)  0.51(0.24-1.06)  0.35(0.15-0.79)  0.44(0.21-0.95) | Age, educational level, beer consumption ,physical activity score height, body mass index at age 18 years, and waist circumference. |
| Liu E  2004, China[^8^](#_ENREF_8) | 1986-2003 | Men living in four small geographically defined areas of urban Shanghai | Cohort | Inci | Self-reported at baseline | Registry | M | 11  7  12  14  9 | *11191156133913101425* | <19.5  19.5-21.1  21.2-22.7  22.8-24.5  >24.6 | 17.25  20.3  21.95  23.65  29.8 | 1.00(ref)  0.70(0.30-1.70)  1.00(0.40-2.20)  1.20(0.50-2.70)  0.70(0.30-1.60) | Age, educational level, and history of emphysema. |
| Pan S  2004, Canada[^9^](#_ENREF_9) | 1994-1997 | Subjects who participated in the NECSS, a collaborative project of Health Canada | PCC | Inci | Self-reported at baseline | Pathology | B | 63  58  75 | 625  569  743 | <25  25-30  ≥30 | 21.5  27.5  32.5 | 1.00(ref)  0.91(0.65-1.29)  1.19(0.76-1.87) | 5-year age group, province of residence, education, alcohol drinking, total caloric intake, vegetable intake, dietary fiber intake, and recreational physical activity. |
| Kubik AK  2004, Czech[^10^](#_ENREF_10) | 1998-2002 | Patients in Prague University Hospital Na Bulovce | HCC | Inci | Self-reported at baseline | Pathology | F | 29  25  37  33 | 215  246  238  323 | <22.9  23.0-25.9  26.0-28.9  >28.9 | 20.7  24.25  27.45  31.95 | 1.00(ref)  0.55(0.30-0.99)  0.72(0.41-1.27)  0.47(0.26-0.83) | Age, residence, education. |
| Kanashiki M  2005, Japan[^11^](#_ENREF_11) | 1993-2003 | Subjects Participating in a  Mass-Screening Program in Ibaraki | PCC | Inci | Measured at the time of diagnosis | Pathology | F  M | 25  20  35  36  1  4  3  4 | 81  106  87  109  33  48  36  42 | <20.8  20.8-22.8  22.9-24.9  ≥25.0  <20.8  20.8-22.8  22.9-24.9  ≥25.0 | 17.9  21.8  23.9  30  17.9  21.8  23.9  30 | 0.80(0.40-1.40)  0.50(0.30-0.90)  1.00(ref)  0.90(0.50-1.50)  0.60(0.10-7.80)  1.00(0.20-6.30)  1.00(ref)  1.80(0.30-11.10) | Age. |
| Kagohashi K  2006, Japan[^12^](#_ENREF_12) | 1987-2005 | Patients in Tsukuba University Hospital | HCC | Inci | Measured at baseline | Pathology | F  M | 67  43  23  34  13  10  5  9 | 131  75  51  66  32  20  10  13 | < 20.8  20.8-22.8  22.9 -24.9  ≥ 25.0  < 20.8  20.8 -22.8  22.9 -24.9  ≥ 25.0 | 17.9  21.8  23.9  30  17.9  21.8  23.9  30 | 1.00(ref)  0.90(0.60-1.40)  1.10(0.60-2.00)  1.00(0.60-1.70)  1.00(ref)  0.80(030-2.20)  0.80(0.20-2.80)  0.60(0.20-1.70) | None. |
| Kabat GC  2007, Canada [^13^](#_ENREF_13) | 1980-2000 | Canadian National Breast Screening Study | Cohort | Inci | Measured at baseline | Registry | F | 12  11  24  23  27 | 5868  5340  11560  10621  12850 | ≤21.6  21.6-23.3  23.3-25.1  25.1-27.9  ≥27.9 | 18.3  22.45  25.6  26.5  31.45 | 1.00(ref)  0.91(0.36-2.31)  1.97(0.88-4.41)  1.81(0.80-4.06)  2.19(1.00-4.80) | Age, education, menopausal status |
| [Kabat GC](http://scholar.google.com.secure.sci-hub.org/citations?user=MVcSR-0AAAAJ&hl=zh-CN&oi=sra)  2008, USA [^14^](#_ENREF_14) | 1998-2006 | Women’s Health Initiative (WHI) Clinical Trial | Cohort | Inci | Measured at baseline | Pathology | F | 42  51  39  30  35 | *8384*  *10396788161206959* | <23.1  23.1-<25.6  25.6-28.3  28.3-32.2  ≥32.2 | 20.8  23.4  26.95  30.25  34.7 | 1.00(ref)  1.24(0.80-1.89)  0.94(0.60-1.50)  0.73(0.45-1.21)  0.83(0.50-1.38) | Age, education, ethnicity, use of hormone, Intakes of total fat, fruits vegetables, alcohol and total calories, physical activity and study |
| Reeves GK  2007, UK[^15^](#_ENREF_15) | 1996-2001 | The Million Women Study | Cohort | Inci | Self-reported at baseline | Registry | F | 269 | 985542 | † per 10 unit increase in BMI | | 0.82(0.59-1.13) | Age, geographical region, socioeconomic status, reproductive history, alcohol intake, physical activity, and, where appropriate, time since menopause and use of hormone replacement therapy. |
| Jee SH  2008, Korea[^16^](#_ENREF_16) | 1992-2006 | Korean Cancer Prevention Study (KCPS) | Cohort | Inci | Measured at baseline | Registry | F  M | NR  799  NR  1767 | NR  160276  NR  415790 | 23-25  >30  NR  >30 | 24  32.5  24  32.5 | 1.00(Ref)  0.84(0.61-1.25)  1.00(Ref)  0.32(0.06-1.22) | Age. |
| Koh WP  2010, Singapore [^17^](#_ENREF_17) | 1993-2006 | Singapore Chinese Health Study | Cohort | Inci | Self-reported at baseline | Registry | B | 23  50  176  38 | 29487  96154  234667  63333 | ≥28  24-28  20-24  <20 | 31.5  26  22  17.5 | 1.00(ref)  0.69(0.42-1.13)  1.01(0.65-1.56)  0.93(0.55-1.56) | Age at baseline, sex, dialect group and year of interview, level of education, and dietary intake of β-cryptoxanthin. |
| Andreotti G  2010, USA[^18^](#_ENREF_18) | 1993-2005 | The Agricultural Health Study | Cohort | Inci | Self-reported at baseline | Registry | B | 1  21  25  3  1 | 313  14373  16816  6459  1884 | <18.5  18.5-24.9  25-29.9  30-34.9  ≥35 | 16.75  21.7  27.45  32.45  37.5 | 2.19(0.053-13.60)  1.00(ref)  1.02(0.55-1.91)  0.32(0.06-1.06)  0.36(0.01-2.26) | State, race, vegetable consumption, exercise. |
| Smith L  2012, USA[^19^](#_ENREF_19) | 1995-2006 | USA National Institutes of Health–AARP Diet and Health Study | Cohort | Inci | Self-reported at baseline | Registry | F  M | 5  54  54  78  41  17  0  16  39  77  29  5 | 9057  154566  176267  259005  119841  652230  2593  64038  162002  317786  90598  21690 | <18.5  18.5-22.49  22.5-24.99  25-29.99  30-34.99  ≥35  <18.5  18.5-22.49  22.5-24.99  25-29.99  30-34.99  ≥35 | 16.75  21.5  23.75  27.5  32.5  37.5  16.75  21.5  23.75  27.5  32.5  37.5 | 1.81(0.72-4.52)  1.17(0.80-1.70)  1.00(ref)  1.00(0.71-1.42)  1.19(0.79-1.80)  1.00(0.58-1.74)  NA  1.04(0.58-1.86)  1.00(ref)  1.01(0.68-1.48)  1.38(0.85-2.24)  1.04(0.41-2.67) | Age at study entry, race/ethnicity, education level, physical activity, and alcohol intake. |
| Bethea  2013, USA[^20^](#_ENREF_20) | 1995-2011 | The Black Women’s Health Study | Cohort | Inci | Self-reported at baseline | Pathology | F | 1  17  12  16 | *573*  *12580*  *9471*  *8918* | <18.5  18.5-24.9  25-29.9  >30 | 16.75  21.7  27.45  32.5 | 1.73(0.23-13.19)  1.00(ref)  0.64(0.30-1.36)  0.83(0.41-1.70) | Age, education, physical activity, alcohol consumption, parity, age at first birth, family history of lung cancer, and geographic region. |
| El-Zein  2013, Canada[^21^](#_ENREF_21) | 1996-2002 | Residents in Greater Montreal who had been diagnosed with lung cancer | PCC | Inci | self-reported 2 years before the diagnosis | Pathology | B | 7  60  47  20 | 18  358  370  110 | <18.5  18.5-24.9  25-29.9  >30 | 16.75  21.7  27.45  32.5 | 2.28 (0.85–6.09)  1.00(ref)  0.70 (0.45–1.08)  1.01 (0.56–1.83) | Age, gender, education, respondent status, ancestry, fruit and vegetable consumption, occupational exposures, recreational physical activity, alcohol |
| Everatt  2014, Lithuania[^22^](#_ENREF_22) | 1978-2008 | Kaunas-Rotterdam Intervention Study (KRIS) and Multifactorial ischemic heart disease prevention study (MIHDPS) | Cohort | Inci | Measured at baseline | Registry | M | 3  13  3 | 412  1159  483 | <25  25-29.9  >30.0 | 21.25  27.5  32.5 | 1.00(ref)  1.69(0.47-6.04)  0.99(0.20-5.00) | Age, alcohol consumption, education, total serum cholesterol, type of cigarettes. |
| Bhaskaran K  2014, UK[^23^](#_ENREF_23) | 1987-2012 | the Clinical Practice Research Datalink (CPRD) | Cohort | Inci | Measured at baseline | Registry | B | NR | 4959307 | * per 5 unit increase in BMI | | 0.99(0.93-1.05) | Age, diabetes status, alcohol use, socioeconomic status, calendar, year, and stratified by sex. |
| Guo L  2014, China[^24^](#_ENREF_24) | 2006-2011 | A large scale population-based cohort study Kailuan Coal Mine in Northern China | Cohort | Inci | Measured at baseline | Pathology | M | 7  94  46  17 | 1763  29355  28730  9314 | <18.5  18.5-24.0  24.0-28  ≥28 | 16.75  21.25  26  31.5 | 1.24(0.57-2.68)  1.00(ref)  0.50(0.35-0.72)  0.57(0.33-0.97) | Age, educational level and alcohol intake. |
| Calle EE  2003, USA[^25^](#_ENREF_25) | 1982-1998 | The Cancer Prevention Study II | Cohort | Mort | Self-reported at baseline | Registry | F  M | 476  224  78  17  156  179  30 | 2544094  1365853  406674  97087  686620  761378  127932 | 18.5–24.9  25.0–29.9  30.0–34.9  35.0-39.9  18.5–24.9  25.0–29.9  30.0–34.9 | 21.7  27.45  32.45  37.45  21.7  27.45  32.45 | 1.00(ref)  0.85(0.73-1.00)  0.99(0.77-1.26)  0.81(0.49-1.31)  1.00(ref)  1.00(0.80-1.24)  0.93(0.63-1.39) | Age, education, physical activity, alcohol use, marital status, race, aspirin use, estrogen replacement therapy , fat consumption, and vegetable consumption. |
| Kondo T  2007, Japan[^26^](#_ENREF_26) | 1988-1999 | The Japan Collaborative Cohort Study for Evaluation of Cancer Risk (JACC Study) | Cohort | Mort | Measured at baseline | Registry | M | 22 | 6,157 | † per 1 unit increase in BMI | | 1.15(1.01-1.32) | Age and family history subjects. |
| Yang  2009, China[^27^](#_ENREF_27) | 1990-2005 | Cohort included 225,721 men recruited from 45 areas throughout China | Cohort | Mort | Measured at baseline | Registry | M | 34  45  105  77  48 | 4294  8625  22480  14331  8068 | <18.5  18.5–19.9  20.0–22.4  22.5–24.9  >25 | 16.75  19.2  21.2  23.7  30 | 1.59(1.12-2.06)  1.08(0.84-1.45)  1.00(ref)  1.19(0.97-1.50)  1.27(0.94-1.75) | Age, study area, self-reported health, status at baseline, education level, alcohol drinking, exposure to indoor and occupational air pollution and dietary intake of fruit and meat. |
| Parr CL  2010,  Asian-Pacific region[^28^](#_ENREF_28) | 1961-1999 | The Asia-Pacific Cohort Studies Collaboration Study | Cohort | Mort | Measured at baseline | Registry | B | 37  267  31  5 | 8990  133524  38005  3393 | <18.5  18.5–24.9  25-29.9  >30 | 16.75  21.7  27.45  32.5 | 2.05(1.18-3.54)  1.00(ref)  0.41(0.26-0.65)  0.71(0.39-1.31) | Study and sex. |
| Leung  2011, China[^29^](#_ENREF_29) | 2000-2008 | The Elderly Health Service clients Cohort in HongKong | Cohort | Mort | Measured at baseline | Registry | B | 24  148  103  142  15 | 1596  13083  9958  15276  2874 | <18.5  18.5-23  23-25  25-30  >30 | 16.75  20.75  24  27.5  32.5 | 1.22 (0.79–1.88)  1.00(ref)  0.94 (0.73–1.21)  0.84 (0.67–1.06)  0.46 (0.27–0.79) | Gender, marital status, education level, housing, alcohol intake and BMI. |

Note: 1. Abbreviations: HCC: hospital-based case-control study; PCC: population-based case-control study; NR: not reported; BMI: body mass index; RRs: relative risks; Inci: incidence; Mort: mortality; F: female; M: male; B: both sex.

2. ‡ The number of cases or participants per category in each study was not always acute, in some studies, the data were not given directly, and we estimated them by the total number of cases and participants. In some cohort studies, the numbers of person-years were used, , In this case, the numbers of participants were equal to the number of person-years divided by follow-up years, and were expressed in italics in the table .

3. When BMI was self-reported, self-administered questionnaire or emailed questionnaire was also taken as the same way.

3. * RR estimates were estimated from the figures presented in the articles, using the software Engauge Digitizer version 2.11 (free software downloaded from <http://sourceforge.net>).

4. † When the RRs were given in other unit, the RRs per 5 kg/m^2^ increase in BMI were calculated by logarithmic transformation.

**References in Table S1:**

1. Knekt P, Heliovaara M, Rissanen A, et al: Leanness and lung-cancer risk. Int J Cancer 49:208-13, 1991

2. Kabat GC, Wynder EL: Body mass index and lung cancer risk. Am J Epidemiol 135:769-74, 1992

3. Drinkard CR, Sellers TA, Potter JD, et al: Association of body mass index and body fat distribution with risk of lung cancer in older women. Am J Epidemiol 142:600-7, 1995

4. Kark JD, Yaari S, Rasooly I, et al: Are lean smokers at increased risk of lung cancer? The Israel Civil Servant Cancer Study. Arch Intern Med 155:2409-16, 1995

5. Xiang Y, Gao Y, Zhong L, et al: [A case-control study on relationship between body mass index and lung cancer in non-smoking women]. Zhonghua Yu Fang Yi Xue Za Zhi 33:9-12, 1999

6. Rauscher GH, Mayne ST, Janerich DT: Relation between body mass index and lung cancer risk in men and women never and former smokers. Am J Epidemiol 152:506-13, 2000

7. Olson JE, Yang P, Schmitz K, et al: Differential association of body mass index and fat distribution with three major histologic types of lung cancer: evidence from a cohort of older women. Am J Epidemiol 156:606-15, 2002

8. Liu E WX, Yuan J, Gao Y: Association of Body Mass Index with Risk of Lung Cancer: Evidence from a Middle-Aged male Cohort in Shanghai, China. Chinese Journal of Clinical Oncology 1:5, 2004

9. Pan SY, Johnson KC, Ugnat AM, et al: Association of obesity and cancer risk in Canada. Am J Epidemiol 159:259-68, 2004

10. Kubik AK, Zatloukal P, Tomasek L, et al: Dietary habits and lung cancer risk among non-smoking women. Eur J Cancer Prev 13:471-80, 2004

11. Kanashiki M, Sairenchi T, Saito Y, et al: Body mass index and lung cancer: a case-control study of subjects participating in a mass-screening program. Chest 128:1490-6, 2005

12. Kagohashi K SH, Kurishima K, Ishikawa H, Ohtsuka M: Body mass index and lung cancer risk in never smokers. Radiol Oncol 40:5, 2006

13. Kabat GC, Miller AB, Rohan TE: Body mass index and lung cancer risk in women. Epidemiology 18:607-12, 2007

14. Kabat GC, Kim M, Hunt JR, et al: Body mass index and waist circumference in relation to lung cancer risk in the Women's Health Initiative. Am J Epidemiol 168:158-69, 2008

15. Reeves GK, Pirie K, Beral V, et al: Cancer incidence and mortality in relation to body mass index in the Million Women Study: cohort study. BMJ 335:1134, 2007

16. Jee SH, Yun JE, Park EJ, et al: Body mass index and cancer risk in Korean men and women. Int J Cancer 123:1892-6, 2008

17. Koh WP, Yuan JM, Wang R, et al: Body mass index and smoking-related lung cancer risk in the Singapore Chinese Health Study. Br J Cancer 102:610-4, 2010

18. Andreotti G, Hou L, Beane Freeman LE, et al: Body mass index, agricultural pesticide use, and cancer incidence in the Agricultural Health Study cohort. Cancer Causes Control 21:1759-75, 2010

19. Smith L, Brinton LA, Spitz MR, et al: Body mass index and risk of lung cancer among never, former, and current smokers. J Natl Cancer Inst 104:778-89, 2012

20. Bethea TN, Rosenberg L, Charlot M, et al: Obesity in relation to lung cancer incidence in African American women. Cancer Causes Control 24:1695-703, 2013

21. El-Zein M, Parent ME, Nicolau B, et al: Body mass index, lifetime smoking intensity and lung cancer risk. Int J Cancer 133:1721-31, 2013

22. Everatt R, Virviciute D, Kuzmickiene I, et al: Body mass index, cholesterol level and risk of lung cancer in Lithuanian men. Lung Cancer 85:361-5, 2014

23. Bhaskaran K, Douglas I, Forbes H, et al: Body-mass index and risk of 22 specific cancers: a population-based cohort study of 5.24 million UK adults. Lancet 384:755-65, 2014

24. Guo L, Li N, Wang G, et al: [Body mass index and cancer incidence:a prospective cohort study in northern China]. Zhonghua Liu Xing Bing Xue Za Zhi 35:231-6, 2014

25. Calle EE, Rodriguez C, Walker-Thurmond K, et al: Overweight, obesity, and mortality from cancer in a prospectively studied cohort of U.S. adults. N Engl J Med 348:1625-38, 2003

26. Kondo T, Hori Y, Yatsuya H, et al: Lung cancer mortality and body mass index in a Japanese cohort: findings from the Japan Collaborative Cohort Study (JACC Study). Cancer Causes Control 18:229-34, 2007

27. Yang L, Yang G, Zhou M, et al: Body mass index and mortality from lung cancer in smokers and nonsmokers: a nationally representative prospective study of 220,000 men in China. Int J Cancer 125:2136-43, 2009

28. Parr CL, Batty GD, Lam TH, et al: Body-mass index and cancer mortality in the Asia-Pacific Cohort Studies Collaboration: pooled analyses of 424,519 participants. Lancet Oncol 11:741-52, 2010

29. Leung CC, Lam TH, Yew WW, et al: Lower lung cancer mortality in obesity. Int J Epidemiol 40:174-82, 2011
