# Supplementary material for: Body mass index and lung cancer risk in never smokers: a meta-analysis
Source: BMC Cancer. 2018 Jun 5;18:635. doi: 10.1186/s12885-018-4543-y (PMC5987408; doi:10.1186/s12885-018-4543-y)
Supplement: Supplementary file 2 — Table S2. Quality scores of the cohort studies included in the meta-analysis, assessed by the Newcastle-Ottawa scale. (DOCX 20 kb) [file 12885_2018_4543_MOESM2_ESM.docx]

| **Author** | **Year** | **Outcome** | **Representativeness of the exposed cohort** | **Selection of the non-exposed cohort** | **Ascertainment of exposure** | **Outcome was not present at start** | **Control for the most important factor (age)** | **Control for any additional factors (education)** | **Assessment of outcome** | **Follow up**  **>5 years** | **Lost rate <20%** | **Overall quality** |
| --- | --- | --- | --- | --- | --- | --- | --- | --- | --- | --- | --- | --- |
| Knekt P | 1991 | Incidence | 1 | 1 | 1 | 0 | 1 | 1 | 1 | 1 | 1 | 8 |
| Drinkard CR | 1995 | Incidence | 1 | 1 | 0 | 1 | 1 | 1 | 1 | 1 | 0 | 7 |
| Kark JD | 1995 | Incidence | 1 | 1 | 1 | 1 | 1 | 0 | 1 | 1 | 1 | 8 |
| Olson JE | 2002 | Incidence | 1 | 1 | 0 | 1 | 1 | 1 | 1 | 1 | 1 | 8 |
| Calle EE | 2003 | mortality | 1 | 1 | 0 | 1 | 1 | 1 | 1 | 1 | 0 | 7 |
| Liu E | 2004 | Incidence | 1 | 1 | 0 | 0 | 1 | 1 | 1 | 1 | 1 | 7 |
| [Kabat GC](http://scholar.google.com.secure.sci-hub.org/citations?user=MVcSR-0AAAAJ&hl=zh-CN&oi=sra) | 2007 | Incidence | 1 | 1 | 1 | 0 | 1 | 1 | 1 | 1 | 1 | 8 |
| Kabat GC | 2007 | Incidence | 1 | 1 | 1 | 0 | 1 | 1 | 1 | 1 | 0 | 7 |
| Reeves GK | 2007 | Incidence | 1 | 1 | 0 | 0 | 1 | 1 | 1 | 1 | 1 | 7 |
| Kondo T | 2007 | mortality | 1 | 1 | 1 | 1 | 1 | 0 | 1 | 1 | 0 | 7 |
| Jee SH | 2008 | Incidence | 1 | 1 | 1 | 1 | 1 | 0 | 1 | 1 | 1 | 8 |
| Yang | 2009 | mortality | 1 | 1 | 1 | 0 | 1 | 1 | 1 | 1 | 0 | 7 |
| Koh WP | 2010 | Incidence | 1 | 1 | 1 | 0 | 1 | 1 | 1 | 1 | 1 | 8 |
| Andreotti G | 2010 | Incidence | 0 | 1 | 1 | 0 | 1 | 0 | 1 | 1 | 0 | 5 |
| Parr CL | 2010 | mortality | 1 | 1 | 1 | 0 | 1 | 0 | 1 | 0 | 0 | 5 |
| Leung | 2011 | mortality | 1 | 1 | 1 | 1 | 1 | 1 | 1 | 1 | 1 | 9 |
| Smith L | 2012 | Incidence | 1 | 1 | 1 | 1 | 1 | 1 | 1 | 1 | 1 | 9 |
| Bethea | 2013 | Incidence | 1 | 1 | 0 | 0 | 1 | 1 | 1 | 1 | 1 | 7 |
| Everatt | 2014 | Incidence | 1 | 1 | 0 | 0 | 1 | 1 | 1 | 1 | 0 | 6 |
| Bhaskaran K | 2014 | Incidence | 1 | 1 | 1 | 1 | 1 | 1 | 1 | 1 | 1 | 9 |
| Guo L | 2014 | Incidence | 1 | 1 | 1 | 0 | 1 | 1 | 1 | 0 | 0 | 6 |

**Table S2 Quality scores of the cohort studies included in the meta-analysis, assessed by the Newcastle-Ottawa scale.**
