# Supplementary material for: Body mass index and lung cancer risk in never smokers: a meta-analysis
Source: BMC Cancer. 2018 Jun 5;18:635. doi: 10.1186/s12885-018-4543-y (PMC5987408; doi:10.1186/s12885-018-4543-y)
Supplement: Supplementary file 3 — Table S3. Quality scores of the case-control studies included in the meta-analysis, assessed by the Newcastle-Ottawa scale. (DOCX 18 kb) [file 12885_2018_4543_MOESM3_ESM.docx]

**Table S3 Quality scores of the case-control studies included in the meta-analysis, assessed by the Newcastle-Ottawa scale.**

| **Author** | **Year** | **Outcome** | **Case definition** | **Representative-ness of the cases** | **Selection of Controls** | **Definition of Controls** | **Control for the most important factor（age）** | **Control for any additional factors (education)** | **Ascertainment of exposure** | **Same method for cases and controls** | **Non-Response Rate <20%** | **Overall quality** |
| --- | --- | --- | --- | --- | --- | --- | --- | --- | --- | --- | --- | --- |
| Kabat GC | 1992 | Incidence | 1 | 1 | 0 | 0 | 1 | 1 | 0 | 1 | 0 | 5 |
| Xiang | 1999 | Incidence | 1 | 1 | 1 | 0 | 1 | 1 | 1 | 1 | 0 | 7 |
| Rauscher GH | 2000 | Incidence | 1 | 1 | 1 | 0 | 1 | 1 | 0 | 1 | 0 | 6 |
| Pan S | 2004 | Incidence | 1 | 1 | 1 | 0 | 1 | 1 | 0 | 1 | 0 | 6 |
| Kubik AK | 2004 | Incidence | 1 | 1 | 0 | 0 | 1 | 1 | 1 | 1 | 1 | 7 |
| Kanashiki M | 2005 | Incidence | 1 | 1 | 1 | 1 | 1 | 0 | 1 | 1 | 0 | 8 |
| Kagohashi K | 2006 | Incidence | 1 | 1 | 0 | 0 | 0 | 0 | 1 | 1 | 0 | 4 |
| El-Zein | 2013 | Incidence | 1 | 1 | 1 | 0 | 1 | 1 | 1 | 1 | 0 | 7 |
